# Supratracheal laryngectomy: a multi-institutional study^☆^

**DOI:** 10.1016/j.bjorl.2019.04.004

**Published:** 2019-05-23

**Authors:** Ariana M. Garcia, Fernando L. Dias, Antônio J. Gonçalves, Claudio R. Cernea, Emilson Q. Freitas, Marcelo B. Menezes, Marco Aurélio V. Kulcsar

**Affiliations:** a Instituto Nacional de Câncer (INCA), Serviço de Cirurgia de Cabeça e Pescoço, Rio de Janeiro, RJ, Brazil; b Irmandade Santa Casa de Misericórdia de São Paulo (ISCMSP), Departamento de Cirurgia de Cabeça e Pescoço, São Paulo, SP, Brazil; c Universidade de São Paulo (USP), Hospital das Clínicas (HC), Departamento de Cirurgia de Cabeça e Pescoço, São Paulo, SP, Brazil

**Keywords:** Supratracheal laryngectomy, Tracheohyoido-epiglottopexy, Tracheohyoidopexy, Laringectomia supratraqueal, Traqueohioidoepiglotopexia, Traqueohioidopexia

## Abstract

**Introduction:**

Supratracheal laryngectomy has been described as a surgical procedure for glottic or supraglottic cancer extending to the subglottic region and/or involving the cricoarytenoid joint, aiming to preserve laryngeal function (breathing, phonation and swallowing), without diminishing locoregional cancer control. The choice of supracricoid laryngectomy in these cases could result in a high risk of compromised resection margins.

**Objective:**

To determine the safety, viability, adequacy of surgical margins and the supratracheal laryngectomy results for intermediate and advanced laryngeal cancer by reviewing the results at three different institutions in Brazil.

**Methods:**

This is a retrospective study that analyzed the charts of 29 patients submitted to supratracheal laryngectomy from October 1997 to June 2017. The type of laryngectomy performed was classified according to the European Laryngological Society classification for horizontal laryngectomies. Early and late results were evaluated. Survival rates (overall, specific, disease-free and total laryngectomy-free survival) were calculated. The mean follow-up time was 44 months.

**Results:**

Of the 29 patients submitted to supratracheal laryngectomy, 25 had no previous treatment. One patient (3.4%) had compromised margins. Four patients (13.8%) had recurrence. Of these, three had local recurrence and one had regional recurrence. Five patients (17.2%) required a total laryngectomy, two due to ruptured pexy and three due to local recurrence. Four of these patients (80%) achieved a successful total procedure. Four patients (13.8%) died, two due to postoperative complications and two due to recurrence. Overall, specific, disease-free and total laryngectomy-free survival at 5 years were, respectively, 82.1%; 88.2%; 83.0% and 80.2%.

**Conclusion:**

Selected patients with intermediate and advanced laryngeal cancer may benefit from supratracheal laryngectomy, that resulted in total laryngectomy-free survival and specific survival of 80.2% and 88.2%, respectively.

## Resumo

**Introdução:**

A laringectomia supratraqueal tem sido descrita como um procedimento cirúrgico com objetivo de preservar a função da laringe (respiração, fonação e deglutição), sem prejuízo no controle oncológico locorregional, para câncer glótico ou supraglótico com extensão à subglote e/ou envolvimento da articulação cricoaritenóidea. A opção pela laringectomia supracricoide nesses casos poderia resultar em grande risco para margens de ressecção comprometidas.

**Objetivo:**

Determinar a segurança, viabilidade, adequação das margens cirúrgicas e os resultados da laringectomia supratraqueal para o câncer de laringe intermediário e avançado através da revisão dos resultados de três instituições distintas no Brasil.

**Método:**

Estudo retrospectivo, com análise dos prontuários de 29 pacientes submetidos à laringectomia supratraqueal, de outubro de 1997 a junho de 2017. O tipo de laringectomia realizada foi classificado de acordo com a classificação da Sociedade Laringológica Europeia para laringectomias horizontais. Foram avaliados os resultados precoces e tardios. As taxas de sobrevida (global, específica, livre de doença e livre de laringectomia total) foram calculadas. O tempo médio de seguimento foi 44 meses.

**Resultados:**

Dos 29 pacientes submetidos à laringectomia supratraqueal, 25 não tinham tratamento prévio. Um paciente (3,4%) teve margens comprometidas. Quatro pacientes (13,8%) recidivaram. Desses, três tiveram recidiva local e um apresentou recidiva regional. Cinco pacientes (17,2%) necessitaram de totalização da laringectomia, duas por ruptura da pexia e três por recidiva local. Quatro desses pacientes (80%) obtiveram sucesso na totalização. Quatro pacientes (13,8%) foram a óbito, dois por complicações pós-cirúrgicas e dois por recidiva. As sobrevidas global, específica, livre de doença e livre de laringectomia total em 5 anos foram, respectivamente, 82,1%; 88,2%; 83,0% e 80,2%.

**Conclusão:**

Pacientes selecionados com câncer intermediário e avançado de laringe podem ser beneficiados com laringectomia supratraqueal, que ofereceu sobrevida livre de laringectomia total e sobrevida específica de 80,2% e 88,2%, respectivamente.

## Introduction

Advanced laryngeal cancer treatment remains challenging for control of disease and preservation of organ function. The conventional surgical therapy for these cases is total laryngectomy, with or without adjuvant radiotherapy.^1^, ^2^ Since total laryngectomy results in loss of function and a high degree of morbidity, organ preservation protocols with chemoradiotherapy have been proposed, with the intent of preserving laryngeal function and achieving a global survival rate of around 60%.^3^, ^4^, ^5^

Another therapeutic option for selected cases with stages III and IV laryngeal tumors is Supracricoid Laryngectomy (SCL); however, a significant number of patients cannot be safely treated by this technique, when there is subglottic extension >1 cm or when the lesion affects the posterior portion of the cricoarytenoid joint.^6^

Supratracheal laryngectomy (STL) has been described as a surgical procedure aimed at preserving laryngeal function (breathing, phonation and swallowing), without affecting locoregional cancer control, for glottic or supraglottic cancer extending to the subglottic area and/or cricoarytenoid joint involvement.

The clinical feature most often characterising these tumors is unilateral vocal cord and arytenoid fixation with cricoarytenoid joint and cricothyroid space involvement, combined with arytenoid and/or cricoid sclerosis. The choice of a SCL in these cases would result in greater risk of positive margins.^6^, ^7^

Indications for STL include glottic tumors with anterior and/or lateral subglottic extension and cricoid ring involvement (T2–T3); glottic and/or supraglottic tumors with paraglottic space invasion and involvement of up to one cricoarytenoid unit, characterized by arytenoid fixation (T3); and locally advanced laryngeal tumors with anterior extension through the thyroid cartilage and minimal extralaryngeal extravasation (T4a). Local contraindications include tumors involving the two arytenoids, the interarytenoid space, the base of the tongue, the hypopharynx and/or the trachea, lesions with gross invasion of the pre-epiglottic space and involvement of the hyoid bone, as well as lesions with large extralaryngeal extravasation.^6^, ^8^, ^9^, ^10^

In 2014, the European Laryngological Society proposed a systematic classification for open partial horizontal laryngectomies, identifying three types of surgical procedures based on the lower limit of resection: Type I, supraglottic laryngectomy; Type II, supracricoid laryngectomy and Type III, supratracheal laryngectomy. For Types II and III, the suffix “a” means that the suprahyoid epiglottis has been preserved and a cricohyoidoepiglottopexy (Type IIa) or tracheohyoidoepiglottopexy (Type IIIa) was performed, while the suffix “b” indicates that the suprahyoid epiglottis has been removed, with the construction of a cricohyoidopexy (Type IIb) or a tracheohyoidopexy (Type IIIb). Additionally, each type of laryngectomy may be extended to adjacent structures. Thus, the surgical resection extension is indicated by the following abbreviations: +ARY, the when resection involves an arytenoid; +BOT, when there is involvement of the base of the tongue; +PIR, for resection of a pyriform sinus; +CAU, in the involvement of a cricoarytenoid unit, consisting of the arytenoid, cricoarytenoid joint and corresponding half of the posterior cricoid lamina.^11^

The aim of this study was to determine the safety, viability, adequacy of surgical margins and the STL results for intermediate and advanced laryngeal cancer, by reviewing the results of three different institutions in Brazil.

## Methods

In the present retrospective study, 29 patients undergoing STL were studied, of whom 14 were from “ISCMSP”, 10 from “INCA” and 5 from “HCFMUSP”, from October 1997 to June 2017. The follow-up period ranged from 2 to 232 months (mean of 44 months).

Among them, 26 (89.7%) were males and 3 (10.3%) were females. Age ranged from 35 to 82 years (mean age of 59 years).

Four patients (13.8%) had undergone previous treatment, with two of them having been submitted to laser surgery, one to radiotherapy and one to cordectomy (Table 1).

**Table 1:** Characteristics of the 29 patients submitted to STL.

	*n*/*N*	%
**Characteristic**		
*Gender*		
*Male*	26/29	89.7
*Female*	3/29	10.3
*Age*		
*Mean (years)*	59	–
*Range (years)*	35–82	–

*Previous treatment*		
*Laser surgery*	2/29	6.9
*Radiotherapy*	1/29	3.4
*Cordectomy*	1/29	3.4

STL = Supratracheal laryngectomy

Regarding tumor location, 27 (93.1%) were in located in the glottic and 2 (6.9%) in the supraglottic area. All cases were reclassified according to the TNM *Union Internationale Contre le Cancer* Classification of Malignant Tumors (UICC-2016). According to the clinical staging, 22 patients (75.9%) were classified as cT3 and 3 (10.3%) as cT4a. Of the patients with previous treatment and disease recurrence, one patient (3.4%) was rT2 (laser surgery) and three (10.3%) were rT3 (radiotherapy, laser surgery and cordectomy). However, pathologically, one patient (3.4%) had no neoplasia, four (13.8%) were pT2, 14 (48.3%) were pT3, and 10 (34.5%) were pT4a. Thus, 4 patients presented a downgrade and 8 presented an upgrade in the pathological staging when compared to the clinical staging. One patient (3.4%) had cN2b neck metastasis at the clinical examination; however, the pathological analysis showed 4 patients (13.8%) with neck metastasis, 2 (6.9%) pN1, 1 (3.4%) pN2a and 1 (3.4%) pN2b. Only one patient (3.4%) was not submitted to neck dissection (Table 2).

**Table 2:** 2016 UICC TNM.

TNM	pT0	pT1	pT2	pT3	pT4a
cT3	0	0	2	12	8
cT4a	0	0	1	0	2
rT2	0	0	1	0	0
rT3	1	0	0	2	0

UICC TNM = TNM Union Internationale Contre le Cancer Classification of Malignant Tumors

The type of surgery performed, according to the European Laryngological Society classification^11^ was IIIa in 4 (13.8%), IIIa + CAU in 17 (58.6%), IIIb in 5 (17.2%) and IIIb + CAU in 3 patients (10.3%) (Table 3).

**Table 3:** Type of surgery according to tumor location.

	Glottic region *n* (%)	Supraglottic region *n* (%)
IIIa	4 (13.8)	0
IIIa + CAU	17 (58.6)	0
IIIb	3 (10.3)	2 (6.9)
IIIb + CAU	3 (10.3)	0

CAU = cricoarytenoid unit

One patient (3.4%) showed compromised resection margins. Regarding adjuvant therapy, 7 patients (24.1%) were submitted to radiotherapy alone and 5 (17.2%) to chemotherapy combined with radiotherapy.

Overall, specific, disease-free and total laryngectomy-free survival was estimated by the Kaplan–Meier method.

## Results

**Figure 1:**
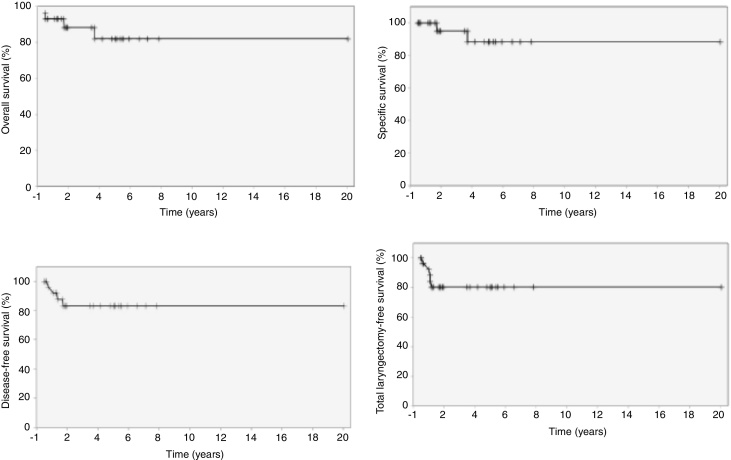
Overall, specific, disease-free and total laryngectomy-free survival at 5 years.

In 5 years, the overall survival rate was 82.1%; the specific survival was 88.2%; the disease-free survival was 83.0%, and 4 patients had recurrence in the first 18 months; the total laryngectomy-free survival was 80.2%, and the 5 cases were submitted to total laryngectomy in the first 12 months after STL (Fig. 1).

One patient (3.4%) showed absence of neoplasia in the STL histopathological report. This patient had mucosal melanoma with cT3N0M0 staging, had been previously submitted to two laser microsurgeries, with compromised margins, but had no residual disease in the salvage STL.

Eight patients (27.6%) had acute postoperative complications, three with neck bleeding, two with ruptured pexy, one with aspiration pneumonia, one with neck cellulitis and one with stroke.

Nine patients (31%) had late complications after treatment, which included two with chronic aspiration, five with laryngeal remnant stenosis, one with aspiration pneumonia and one with dyspnea due to redundant laryngeal mucosa. Patients with chronic aspiration were undergoing speech-language therapy. Those who developed laryngeal stenosis maintained the use of the tracheotomy cannula. The patient with aspiration pneumonia was treated only with antibiotic therapy, since he was already in palliative care due to recurrent and unresectable neck disease. The patient with redundant laryngeal mucosa was successfully treated with endoscopic laser resection (Table 4).

**Table 4:** Acute and late postoperative complications.

	*n*/*N*	%
*Acute complications*		
Neck bleeding	3/29	10.3
Ruptured pexy	2/29	6.9
Aspiration pneumonia	1/29	3.4
Neck cellulitis	1/29	3.4
Stroke	1/29	3.4
Total	8/29	27.6

*Late complications*		
Chronic aspiration	2/29	6.9
Neolarynx stenosis	5/29	17.2
Aspiration pneumonia	1/29	3.4
Dyspnea	1/29	3.4
Total	9/29	31

Four patients (13.8%) had disease recurrence. Of these, three had local recurrence (one pT3N0M0 patient who was submitted to adjuvant radiotherapy had recurrence after 9 months; one pT3N0M0 patient who had the margins compromised in STL, underwent adjuvant chemoradiotherapy and had recurrence after 4 months; a pT3N0M0 patient previously treated with radiotherapy, submitted to salvage STL, showed recurrence after 7 months) and one had regional recurrence (pT3pN2b, submitted to adjuvant chemoradiotherapy, had an unresectable neck recurrence and died after 15 months).

Five patients (17.2%) required total laryngectomy, 2 (6.9%) due to ruptured pexy and 3 (10.3%) due to local disease recurrence. Four of these patients (80%) had a successful salvage total laryngectomy.

Four patients (13.8%) died, among them two due to postoperative complications (one due to neck bleeding on the 5th postoperative day and one due to a stroke on the 11th postoperative day) and two due to regional unresectable disease recurrence (one died 15 months after the STL and one had local recurrence 9 months after the STL, was submitted to total laryngectomy, developed neck metastasis and died after 30 months).

Twenty-five of 27 patients (92.6% – excluding the two who died due to acute complications in the postoperative period) were able to receive an oral diet, and the time of withdrawal of the feeding tube ranged from 23 to 397 days (median of 45 days); one patient (3.7%) who was operated 9 months ago, was still dependant on enteral feeding due to chronic aspiration by the time this work was written and one patient (3.7%) who had an unresectable cervical recurrence, had to receive diet through a gastrostomy tube until death.

Regarding the tracheotomy, 16 of 22 patients (72.7% - excluding the two who died due to postoperative complications and five submitted to total laryngectomy) were decannulated, and the time of decannulation ranged from 11 to 257 days (median of 50 days). Five patients (22.7%) remained with tracheotomy due to stenosis of the laryngeal remnant and 1 (4.6%) due to chronic aspiration.

The rate of laryngeal function preservation, regarding the ability to feed without a feeding tube, comprehensible speech and breathing without a tracheotomy, was 68.2%. Of the 22 patients (excluding the two who died due to complications in the postoperative period and five who underwent total laryngectomy), five patients were still tracheotomy-dependant, 1 patient mantained the use of enteral feeding, and 1 patient had tracheotomy plus enteral feeding.

Table 5 shows the analysis of pathological staging, acute and late complications, total laryngectomy, deaths and evaluation of patients who withdrew enteral feeding and who were decannulated per institution.

**Table 5:** Analysis by institution.

	“ISCMSP” *n* (%)	“INCA” *n* (%)	“HCFMUSP” *n* (%)
*N*	14 (48.3)	10 (34.5)	5 (17.2)

*pT*			
pT0	0	1 (3.4)	0
pT1	0	0	0
pT2	0	1 (3.4)	3 (10.3)
pT3	7 (24.1)	7 (24.1)	0
pT4a	7 (24.1)	1 (3.4)	2 (6.9)

*Acute complications*			
Neck bleeding	2 (6.9)	1 (3.4)	0
Ruptured pexy	1 (3.4)	0	1 (3.4)
Aspiration pneumonia	0	1 (3.4)	0
Neck cellulitis	0	1 (3.4)	0
Stroke	1 (3.4)	0	0

*Late complications*			
Chronic aspiration	2 (6.9)	0	0
Neolarynx stenosis	5 (17.2)	0	0
Aspiration pneumonia	0	1 (3.4)	0
Dyspnea	0	1 (3.4)	0

*Total laryngectomy*			
Ruptured pexy	1 (3.4)	0	1 (3.4)
Local recurrence	2 (6.9)	1 (3.4)	0

*Causes of death*			
Neck bleeding	1 (3.4)	0	0
Stroke	1 (3.4)	0	0
Disease recurrence	1 (3.4)	1 (3.4)	0

*Enteral feeding*			
Withdrew	11/12 (91.7)	9/10 (90)	5/5 (100)
Range of time of use (days)	30–330	23/397	30–203

*Tracheotomy*			
Decannulated	3/9 (33.3)	9/9 (100)	4/4 (100)
Range of time of use (days)	47–257	11–90	60–230

In the last follow-up, of the 25 patients who survived, none had locoregional disease recurrence and 1 (4%) was followed due to an isolated pulmonary nodule with no diagnostic definition so far.

## Discussion

The first STL with tracheohyoidoepiglottopexy was performed by Serafini in 1972. This procedure involved the preservation of the suprahyoid epiglottis and pexy between the hyoid bone, the remaining epiglottis and the first tracheal ring; however, this technique removed the two arytenoids, with a poor functional result. Therefore, it was abandoned in the early 1980s.^12^

In 1990, Laccourreye et al. published the oncological and functional results of SCL with cricohyoidoepiglottopexy for glottic cancer, performed in 36 patients. In 34 cases, both arytenoids were preserved; however, in one patient, the cricoarytenoid joint was disarticulated with resection of one arytenoid, and in another, resection of the anterolateral portion of the cricoid cartilage with trachecricohyoido-epiglottopexy was performed. No complications occurred in these two patients, and both were decannulated at 7 and 3 days, respectively. The 3-year survival rate was 86.5% and local and regional recurrence rates were 5.6% and 8.4%, respectively. In this study, all the patients were decannulated (mean time 7 days, 3–57 days), recovered the swallowing and withdrew enteral tube (mean time of 15 days, 9–30 days) and had vocal quality that allowed social interaction.^13^

In another publication, the same authors describe the oncological and functional results of SCL with cricohyoidopexy for supraglottic/transglottic tumors, performed in 68 patients. In this study, a 3-year survival rate of 71.4% was observed, with no local recurrence, but with a regional recurrence rate of 5.8% and a distant metastasis rate of 8.8%. All patients were decannulated (mean time of 7 days, 3–51 days); 74.6% acquired normal swallowing and withdrew the enteral tube (mean time of 15 days, 13–70 days) and all had phonation that allowed social interaction. One patient died on the 3rd postoperative day due to rupture of an abdominal aortic aneurysm and another in the 5th month due to recurrent aspiration pneumonia.^14^

In 104 patients submitted to SCL with cricohyoidoepiglottopexy between 1972 and 1985, Piquet and Chevalier achieved an overall survival rate at 5 years of 75%, withdrawal of the enteral tube within 45 days postoperatively in 100% of cases and decannulation in up to 28 days of 81.5% of the patients.^15^

In 1996, Laccourreye et al. described a modification in the conventional SCL technique, in which the cricoid ring was removed in cases of glottic tumors with anterior subglottic extension. In this series of 21 patients, survival rates, local control, neck recurrence, and distant metastasis at 5 years were, respectively, 74.7%; 88.9%; 11.1% and 22.4%, with a laryngeal preservation rate of 90.5%.^16^

In 1997, another study published by Laccourreye et al. described the cases of ruptured pexy after SCL. During a period of 22 years (1974–1996), the incidence of this complication was 0.8% (3/371). In these situations, one may choose to perform a total laryngectomy or to review the pexy, with resection of the anterior cricoid arch and reconstruction with trachecricohyoido-epiglottopexy.^17^ In our series, the 2 cases of ruptured pexy were treated with total laryngectomy.

In the study by Lima et al., which evaluated the functional and oncologic results of 43 patients with T3/T4 glottic cancer treated with SCL with cricohyoidoepiglottopexy, specific survival and disease-free survival of 78% and 83% were observed at 5 years, respectively. The rate of laryngeal preservation was 83.7%.^1^ In a previous study by the same authors, 2 of 27 patients submitted to SCL with cricohyoidoepiglottopexy due to T2/T3 glottic cancer required total laryngectomy (one due to recurrent aspiration pneumonia and one due to ruptured pexy).^18^

In 2006, Rizzotto et al. proposed a change in the previously described extended SCL technique that allowed preservation of function and the possibility of postoperative rehabilitation. This technique is based on the resection of the entire glottis and part of the subglottis, in addition to the thyroid cartilage, preserving both or at least one functioning cricoarytenoid unit, consisting of the arytenoid, cricoarytenoid joint and corresponding half of the posterior cricoid lamina. Inferiorly, the resection limit encompasses the entire cricoid ring and half of the posterior lamina, preserving the first tracheal ring. The type of reconstruction, tracheohyoidoepiglottopexy or tracheohyoidopexy, differs according to the supraglottic resection extent.^8^, ^9^

Subsequently, these authors evaluated the oncological and functional results of 115 patients submitted to STL between 2002 and 2011. The overall, disease-free survival and locoregional control in 3 years were, respectively, 84.6%; 72.3% and 73.3%; and, in 5 years, 78.9%; 68.5% and 69.6%. The nasoenteral tube or gastrostomy was withdrawal in 97.4% of the patients, with an average time of 21 days (12–161 days). The mean tracheotomy occlusion time was 86 days (29–489 days).^19^

In our series, one patient underwent SCL that extended to the posterolateral portion of the cricoid cartilage, with the resection of a cricoarytenoid unit in 1997, when STL had not yet been described. This case was reclassified as type IIIa + CAU laryngectomy and included in the study. All other cases were performed after the technique description.

Succo et al. published the results of the largest series in the international literature, with 142 patients submitted to STL in a multi-institutional study carried out in Italy. In this, 21.1% of the cases had disease recurrence, being 70% local recurrence and 30% regional recurrence. The overall, specific, disease-free and total laryngectomy-free survival at 5 years were, respectively, 78.7%; 90.4%; 69.1% and 85.4%.^6^

The oncological results of the present study are comparable to those published in the international literature to date, with overall, specific, disease-free and total laryngectomy-free survival at 5 years of 82.1%, 88.2%, 83.0% and 80.2%, respectively.

A study by Rizzotto et al. evaluated 469 patients submitted to SCL (399) and STL (70) during a period of over 10 years and compared oncological and functional results between them. The overall and disease-free survival at 5 years and preservation of laryngeal function after 2 years of surgery were, respectively, 95.6%; 90.9% and 95.7% for SCL and 80%; 72.9% and 80% for STL. The total laryngectomy rate among all patients in this series was 4.4%. Withdrawal of the nasoenteral tube or gastrostomy occurred in 99.4% of the patients (mean time of 16 days, 4–161 days) and decannulation occurred in 98.3% of the cases (mean time of 73 days, 16–852 days). Both types of laryngectomies showed high survival rates than those obtained through organ preservation protocols, based on chemoradiotherapy.^20^ In our series, the functional result was slightly lower, with 92.6% of the patients withdrew the enteral feeding (median of 45 days, 23–397 days) and 72.7% were decannulated (median 50 days, 11–257 days).

Regarding the acute and late postoperative complications, we observed neck bleeding, neck cellulitis, laryngeal remnant stenosis, dyspnea and aspiration pneumonia in 10.3%; 3.4%; 17.2%; 3.4% and 6.9% of cases, respectively. In the study by Rizzotto et al., these rates were 1.7%; 0.9%; 17.4%; 0.9% and 9.6%; and in the study by Succo et al., they were 0.7%; 1.4%; 17.6%; 2.1% and 9.9%.^6^, ^19^

The long-term functional results after STL have been described in the international literature. A study published in 2015 evaluated swallowing, voice and quality of life in a group of 22 patients submitted to this procedure. Swallowing recovery was achieved in 20 cases, with two patients showing severe dysphagia for solid foods. The voice in the postoperative period was highly dysphonic and the maximum phonation time was significantly reduced, but it was enough to allow normal spontaneous speech. Despite that, patients reported only a small impact on their quality of life. Mild dysphagia and aspiration pneumonia represent the most common, although infrequent, early complications after this procedure and are usually well tolerated. Laryngeal remnant stenosis is the most common late complication. The functional results of STL are similar to those observed with SCL.^9^, ^21^, ^22^, ^23^

The expansion of the STL lower limit increased the SCL indications, breaking some paradigms. Cases involving one cricoarytenoid unit and/or with subglottic extension were considered contraindications for SCL, and total laryngectomy was indicated. With the advent of this technique, these selected cases were treated with subtotal laryngectomy, with laryngeal function preservation and no harm to locoregional disease control.

## Conclusion

Selected patients with intermediate and advanced laryngeal cancer may benefit from STL, which in this study resulted in total laryngectomy-free and specific survival of 80.2% and 88.2%, respectively.

## Conflicts of interest

The authors declare no conflicts of interest.
